# New Approaches of Generalised Fuzzy Soft sets on fuzzy Codes and Its Properties on Decision-Makings

**DOI:** 10.12688/f1000research.158747.2

**Published:** 2025-04-09

**Authors:** Masresha Wassie Woldie, Jejaw Demamu Mebrat, Mihret Alamneh Taye

**Affiliations:** 1 Bahir Dar University Department of Mathematics, Bahir Dar, Amhara, Ethiopia; 2 Debark University, Debark, Amhara, Ethiopia; 3 Bahir Dar University Department of Mathematics, Bahir Dar, Amhara, Ethiopia

**Keywords:** Fuzzy Soft set, Fuzzy code, interval-valued fuzzy soft code, generalised fuzzy soft set, Generalised fuzzy soft code.

## Abstract

**Background:**

Several scholars defined the concepts of fuzzy soft set theory and their application on decision-making problem. Based on this concept, researchers defined the generalised fuzzy soft set and its applications. However, to the best of the author’s knowledge, the generalised fuzzy soft set has not been dealing with in the generalised fuzzy soft code set. In this paper, we introduce the notion of generalised fuzzy soft code set and its application.

**Methods:**

The theory of fuzzy soft sets and its application, generalised fuzzy soft sets and fuzzy codes in different years were studied with various researchers. To derive a generalised fuzzy soft code set, we apply the concepts of generalised fuzzy soft set and a new view of fuzzy codes and its application. A new aspect of this paper is to introduce the definition of generalised fuzzy soft code sets and its application on medical diagnosis and decision-makings.

**Results:**

Generalised fuzzy soft code is the most powerful and effective extension of fuzzy soft sets that deal with the choice’s parameterized values. It is an extended model of fuzzy soft sets and a new mathematical tool with significant advantages for handling uncertain information and is proposed by combining the concept of fuzzy soft sets and fuzzy code sets. This paper introduces the concept of generalised fuzzy soft code and its properties.

**Conclusions:**

In this study, we combine generalised fuzzy soft set and a different approach to coding theory to introduce generalisation of fuzzy soft codes. The paper also considers the relation between generalised fuzzy soft code and its application. We discussed the matrix representation of generalised fuzzy soft code. Furthermore, a demonstration example illustrates how the strategy could be effectively applied to various problems.

## 1. Introduction

Zadeh (1965) established the notion of a “fuzzy set” as a way to represent a class of objects with different membership grades. A membership function, also known as a characteristic function, describes such a set, with each item’s membership degree ranging from 0 to 1. Molodtsov (1999) presented the theory of soft sets as a novel mathematical technique for handling uncertainties that are outside the scope of current mathematical techniques. In order to address complicated issues including ambiguity and uncertainty, Molodtsov created a universal mathematical tool that may be applied to both conventional and some modern mathematical methods. Fuzzy sets are conceptualized in a way that makes their methods intuitively clear. Maji, Biswas, and Roy (2003) developed the theory of soft sets. Gogoi, Kr. Dutta, and Chutia (2014) introduced an application of fuzzy soft set theory in day to day problems; Roy and Maji (2007) presented the application of fuzzy soft set theory to decision-making problems. Amudhambigai and Neeraja (2019) discussed a new view of fuzzy codes and its application. Ozkan and Mehmet (2002) introduced different approaches of fuzzy codes and their properties. Malik, Mordeson, and Nair (1992) defined the concept of a fuzzy generating set and describe the fuzzy subgroup which it generates and introduced the notion of a minimal fuzzy generating set. Ali et al. (2018) designed and develop a new class of linear algebraic codes defined as soft linear algebraic codes using soft sets. They also discussed some algebraic properties of soft codes. Garg and Arora (2018) describe the concept of generalized IFSS. Kong, Wang, and Wu (2011) studied the implementation of grey theory-based fuzzy soft sets in decision-making situations. Lin et al. (2018) a set of code words from the cyclic code are decoded using a binary parity-check matrix in a soft-joint manner. Gereme, Demamu, and Alaba (2023), Hamming distance of fuzzy codes and other features of binary fuzzy codes. ( Lin et al. 2018) a novel coding technique for the Galois-Fourier transform domain that is intended for collective encoding and collective iterative soft-decision decoding of cyclic codes of prime lengths. Kamble (2017) examined the codes that emerged from soft sets and fuzzy sets, and using fuzzy linear space, explained the properties of fuzzy linear codes. Kim (2023) developed a fuzzy linear code description based on linear algebraic codes. Tsafack et al. (2018) established the concepts of fuzzy cyclic and linear codes over a Galois ring. Majumdar and Samanta (2010) established generalized fuzzy soft sets and investigated a few of their characteristics. It has been demonstrated that generalized fuzzy soft sets can be used to diagnose medical conditions and decision-making problem. Adde, Toro, and Jego (2012) examined linear block code of maximum likelihood soft-decision decoding. Dauda, Mamat, and Waziri (2015) an application of fuzzy soft sets based on a thorough theoretical analysis of the fundamental operations of soft sets and a definition of soft sets. Kané (2021) suggested a different method for figuring out the fuzzy optimal solution to a semi-fully fuzzy linear programming issue. Ali et al. (2023) discussed the generalized intuitionistic decision-theoretic rough set, a combination of intuitionistic fuzzy sets and decision-theoretic rough sets.

The researchers studied new operational laws for interval-valued Pythagorean fuzzy soft numbers and create two interaction operators-the interval valued Pythagorean fuzzy soft interaction weighted average and the interval-valued Pythagorean fuzzy soft interaction weighted geometric operators, and analysed their properties ( Zulqarnain et al. 2023a). Scholar proposed the correlation coefficient and weighted correlation coefficient for interval valued Pythagorean fuzzy soft set and examine their necessary properties ( Muhammad et al. 2023). Zulqarnain et al (2022a) investigated some average aggregation operators, such as q-rung ortho pair fuzzy soft Einstein weighted average and q-rung ortho pair fuzzy soft Einstein ordered weighted average operators. Zulqarnain et al. (2022b) developed some novel operational laws for q- rung ortho pair fuzzy soft numbers. The scholars introduced T-SF partitioned Bonferroni mean (T-SFPBM) and T-SF weighted partitioned Bonferroni mean (T-SFWPBM) operators to fuse the evaluation information provided by experts. Then, an IFM is designed to achieve a consensus between multiple experts ( Gurmani et al. 2023a). The researchers proposed the concept of interval-valued probabilistic linguistic T-spherical fuzzy set ( Gurmani et al. 2023b). Zulqarnain et al. (2024) introduced the interval-valued q-rung ortho pair fuzzy soft Einstein-ordered weighted and Einstein hybrid weighted aggregation operators. The scholars investigated the formulation of the q-rung ortho pair fuzzy soft Einstein hybrid weighted average operator and its specific characteristics ( Zulqarnain et al. 2023b). The researcher developed a new approach to determining expert weights using distance and similarity measures for interval-valued q-rung ortho pair fuzzy numbers ( Hussain & Harish 2023). The scholars proposed the generalized version of the multipolar neutrosophic soft set with operations and basic properties ( Zulqarnain et al. 2021). Zulqarnain et al. (2022c) some novel operational laws for interval valued Pythagorean fuzzy soft set have been proposed. Zulqarnain et al. (2022d) developed the Einstein-weighted ordered aggregation operators for the Pythagorean fuzzy hyper soft set, which proficiently contracts with tentative and confusing data. The researchers also introduce the Einstein operational laws for Pythagorean fuzzy soft numbers ( Zulqarnain et al. 2022e). The scholars described and evaluated the characteristics of the correlation coefficient (CC) and weighted correlation coefficient (WCC) for q-ROFSS ( Muhammad et al. 2024). Studied the novel Einstein aggregation operators (AOs) for this model, specifically the interval-valued q-rung ortho pair fuzzy soft Einstein weighted average (IVq-ROFSEWA) and interval-valued q-rung ortho pair fuzzy soft Einstein weighted geometric (IVq-ROFSEWG) ( Mukherjee & Mukherjee 2022). Zulqarnain et al. (2022) proposed Einstein’s operational laws for q-rung ortho pair fuzzy soft numbers (q-ROFSNs).

However, as far as the author’s awared, no studied pertaining to the fundamentals of generalised fuzzy soft code have been released. Thus, motivated by the formationed works, the current study seeks to introduce this idea. To derive a generalised fuzzy soft code and its properties, we integrate the concepts of a generalised fuzzy soft set ( Majumdar and Samanta 2010) and a different approaches of coding theory ( Ozkan & Mehmet 2002). A new aspect of this paper is to introduce and develop the definition and application of generalised fuzzy soft code by combining the concepts of fuzzy soft set and fuzzy codes. The definition and features of relations on generalised fuzzy soft codes are proposed.

The organization of this study as follows: Section 2 included fundamental notions and properties of preliminary concepts, Section 3, Generalised fuzzy soft code, and some of their characteristics, whereas, Section 4 takes into consider relation on generalised fuzzy soft codes and its application.

## 2. Methods

In this section, we review a few fundamental concepts that we utilize to get our outcomes.

Definition 1. Ozkan & Mehmet (2002) The notion $F_{q}=\left(a_{1},a_{2},\ldots ,a_{q}\right),$ is a q-ary code which is a collection of symbol sequences in which each symbol is chosen from a set of q different components. The set $F_{q}$ is sometimes referred to as the alphabet and is defined as the set

$$F_{q}=\left\{\bar{0},\bar{1},\bar{2},\ldots ,\bar{q-1}\right\}$$

Definition 2. ( Amudhambigai & Neeraja 2019) $F^{n}$ is the collection of all ordered n− tuples

$p=p_{1},p_{2},\ldots ,p_{k},$ Where each *p*_*i*_ in $F_{q}$ . The elements of $F^{n}$ are referred to as words or vectors.

Definition 3. Ozkan & Mehmet (2002) Let us assume that C has a as a code word. The relative weight of a code word a, denoted as

$R_{w}\left(a\right)$ , is the sum of $p_{1},p_{2},\ldots ,p_{k}$ if $p_{1},p_{2},\ldots ,p_{k}$ are defined as the positions of 1 _s_ in ***a***. For instance, $R_{w}\left(a\right)=1+3+4+5=13$ if a=10111 is a code word of C in $F^{5}$ .

1+2+3+…+n is the relative weight of 111…1, which is a code word of C in $F_{2}^{n}$ . This can be articulated as the sum of the first *n* positive integers. The largest relative weight of code C in $F_{2}^{n}$ is referred to as this matter. Where n is the length of a code word, where $R_{\mathrm{wr}}$ is a mapping defined as $R_{\mathrm{wr}}:C\to \left[0,1\right]$ for ***C*** ∈ *F*_2_^*n*^ and $R_{\mathrm{wr}}\left(a\right)\in \left[0,1\right],$ for any a∈C .

Definition 4. ( Ozkan & Mehmet 2002) Let $\left\{c_{1},c_{2},\ldots ,c_{n}\right\}$ is the code word of C with length *n*. Let C be a code.

For each i=1,2,…,n, let $R_{\mathrm{wr}}\left(c_{i}\right)$ be a fuzzy code word related to the code words $c_{i}$ , $c_{j}$ in C . Then the fuzzy code intersection and union of any two fuzzy codes $R_{\mathrm{wr}}\left(c_{i}\right)$ and $R_{\mathrm{wr}}\left(c_{j}\right)$ , respectively, are given as follows:

(i)
$R_{\mathrm{wr}}\left(c_{i}\right)⋂R_{\mathrm{wr}}\left(c_{j}\right)=\min\left\{R_{\mathrm{wr}}\left(c_{i}\right),R_{\mathrm{wr}}\left(c_{j}\right)\right\}$

(ii)
$R_{\mathrm{wr}}\left(c_{i}\right)⋃R_{\mathrm{wr}}\left(c_{j}\right)=\max\left\{R_{\mathrm{wr}}\left(c_{i}\right),R_{\mathrm{wr}}\left(c_{j}\right)\right\}$

Definition 5. ( Amudhambigai and Neeraja 2019) For any fuzzy code $R_{\mathrm{wr}}\left(c_{i}\right)$ , the complement is $c\left(R_{\mathrm{wr}}\left(c_{i}\right)\right)$ is calculated by subtracting from 1 the relative weight of each member of C .

That is,

$$c\left(R_{\mathrm{wr}}\left(c_{i}\right)\right)=1-R_{\mathrm{wr}}\left(c_{i}\right)$$ (1)

Definition 6. ( Molodtsov 2011) Consider the idea of a set of parameters represented by E and U be the notion of a starting universe of items. P(U) is the power set of U and A⊆E *.* A pair (F,A) is a soft set over U if and only if F is a mapping given by

F:A→P(U)

In other words, the soft set is a parameterized family of subjects from the set *U*.

Consider the set of *e* ∈ *A*, or the set of e− approximate components of the soft set, where F(e) can be arbitrary, some of which can have nonempty intersection, and some of which can be empty. This applies to every *F* ( *e*) from this family for *e* in A . where the attributes, traits, or properties of objects are usually the arguments. We might consider Zadeh’s fuzzy set as a subset of the soft set. Given a fuzzy set A , its membership function is $\mu_{A}$ , that is, *μ*_*A*_ is a mapping from U into [0,1], that is

$$\mu_{A}:U\to \left[0,1\right]$$

Definition 7. ( Majumdar & Samanta, 2010) The set of all fuzzy subsets within a universal set U is denoted by $I^{U}$ .

Assume A⊆E and that E is a parameter set. Then, a pair (F,A) is a fuzzy soft set over U , where F is a mapping denoted by

$$F:A\to I^{U}$$

Definition 8. ( Ali et al. 2018) Assume that a vector space of size n over the field K is represented by $W=K^{n}$ . (F,E) is as of *t* algebraic linear code over K . For every $\;F\left(e_{i}\right);1\leq i\leq t$ , the symbol F(E)=F(e1),…,F(et) in x indicates a linear algebraic code of W . The number of linearly independent elements of $F\left(e_{i}\right)$ is denoted by $d_{i},$ which is the dimension of $F\left(e_{i}\right)$ . Each $F\left(e_{i}\right)\in F\left(E\right)$ in this instance represents a linear algebraic code. The soft dimension of (F,E) is $\left(F,E\right)=\left\{d_{1},d_{2}\ldots ,d_{t}\right\}$ and the number of soft code words of (F,E) is given by:

$$n\left(F,E\right)=|F\left(e_{1}\right)|\times |F\left(e_{2}\right)|\times \ldots \times |F\left(e_{t}\right)|$$ (2)

Where 1≤i≤t .

Keep in mind that dim(F,A) represents the soft dimension (F,E) .

Definition 9. ( Majumdar & Samanta 2010). The universal set of elements is $=\left\{x_{1},x_{2},\ldots ,x_{n}\right\}$ , and the universal set of parameters is $E=\left\{e_{1},e_{2},\ldots ,e_{m}\right\}.$ We shall refer to the pair (U,E) as a soft universe. Let μ be a fuzzy subset of E , that is,

$$\mu :E\to \left[0,1\right],\;\text{and let}\;F:E\to I^{U}.$$

where the set of all fuzzy subsets of U is represented by $I^{U}$ . Assume that $F_{\mu }$ represents the mapping. Let a function Fμ such that

$\mathrm{F\mu}:E\to I^{U}\times I$ have the following definition:

Fμ(e)= F(e),μ(e)) , where $\left(e\right)\in I^{U}$ .

A generalised fuzzy soft set (in short GFSS) over the soft universe (U,E) is then denoted by Fμ . Here, for every parameter $e_{i}$ , $\mathrm{F\mu}\left(e_{i}\right)=\left(F\;\left(e_{i}\right),\mu \left(e_{i}\right)\right)$ showed both the degree of belongingness that $\mu \left(e_{i}\right)$ represents and the degree of belongingness of the elements of U in $F\left(e_{i}\right).$

Definition 10. ( Majumdar & Samanta 2010) Let Fμ and $G_{\sigma }$ be two generalised fuzzy soft sets over (U,E). Now Fμ is said to be a generalised fuzzy soft subset of $G_{\sigma }$ if

(i)
μ⊆σ

(ii)
F(e)⊆G(e) , for all e∈E , in this case we write F(e) is a fuzzy subset of G(e) .

Definition 11. ( Majumdar and Samanta 2010) Consider a generalised fuzzy soft set over (U,E) , denoted by Fμ . Then, $F^{∁}$ represents the complement of Fμ , which is defined as

$$F^{∁}=G_{\sigma },\text{where}\;G\left(e\right)=F^{∁}\;\text{and}$$

$$\sigma \left(e\right)=\mu^{∁}\left(e\right),\;\text{for all}\;e\in E.$$

Definition 12. ( Majumdar & Samanta 2010) $\mathrm{F\mu}⋃G_{\sigma }$ represents the union of two generalised fuzzy soft sets Fμ and $G_{\sigma }$ . This results in a generalised fuzzy soft set $H_{\nu }$ , which is defined as $H_{\nu }:E\to I^{U}\times I$ such that $H_{\nu }\left(e\right)=\left(H\left(e\right),\nu \left(e\right)\right)$ where H(e)=F(e)°G(e),ν(e)=μ(e)°σ(e), and ° denotes a t-co norm.

Definition 13. ( Majumdar & Samanta 2010) $\;\mathrm{F\mu}⋃G_{\sigma }$ represents the intersection of two generalised fuzzy soft sets, Fμ and $G_{\sigma }$ . This leads to a generalised fuzzy soft set $H_{\nu }$ , which is defined as

$H_{\nu }:E\to I^{U}\times I$ such that $H_{\nu }\left(e\right)=\left(H\left(e\right),\nu \left(e\right)\right)$ , where ∗ is a t-co norm and

H(e)=F(e)∗G(e)andν(e)=μ(e)∗σ(e).

Definition 14. ( Majumdar & Samanta 2010) $\emptyset_{\theta }:E\to I^{U}\times I$ indicates a generalised null fuzzy soft set, which is referred to as a generalised fuzzy soft set, such that $\emptyset_{\theta }\left(e\right)=\left(F\;\left(e\right),\theta \left(e\right)\right),$ where $\emptyset \left(e\right)=\bar{0}$ (null set or no element is included under any parameter within the soft set), and, θ(e)=0 , for all e∈E .

Definition 15. ( Majumdar & Samanta 2010) A generalised absolute fuzzy soft set, represented by

$A_{\alpha }:E\to I^{U}\times I,$ is refered to as a generalised fuzzy soft set, where

$A_{\alpha }\left(e\right)=F\;\left(e\right),\alpha \left(e\right)$ is defined by $A\left(e\right)=\bar{1}$ (maximal soft set or where every parameter is associated with the whole set under consideration) and α(e)=1 , for all e∈E *.*

## 3. Results

### 3.1 Generalised Fuzzy Soft Code (GFSC)

In this subsection, we introduce generalised fuzzy soft codes and its properties.

Definition 16. Let $U=R_{\mathrm{wr}}\left(a_{i}\right)$ for which C is the universal element, consider the set of parameters be E, and $a_{i}$ is an element of a code vector. The fuzzy soft code universe is a pair (F,E).

Let $F:E\to P\left(R_{\mathrm{wr}}\left(a_{i}\right)\right),$ where all fuzzy code subsets of $R_{\mathrm{wr}}\left(a_{i}\right)\in \left[0,1\right]$ are collected as $P\left(R_{\mathrm{wr}}\left(a_{i}\right)\right)$ . And consider a mapping μ and $F_{\mu }$ such that

$$\mu :E\to R_{\mathrm{wr}}\left(a_{i}\right)$$ (12)

is a fuzzy subset of E and

$$\;F_{\mu }:E\to P\left(R_{\mathrm{wr}}\left(a_{i}\right)\right)\times R_{\mathrm{wr}}\left(a_{i}\right)$$ (13)

defined as $F_{\mu }\left(e_{i}\right)=\left(F\left(e_{i}\right),\mu \left(e_{i}\right)\right),\forall e\in E$ and $F\left(e_{i}\right)\in P\left(R_{\mathrm{wr}}\left(a_{i}\right)\right)$ . Then, $F_{\mu }$ is called a generalised fuzzy soft code (GFSC) over the soft universe.

$\left(R_{\mathrm{wr}}\left(a_{i}\right),E\right).\mu \left(e_{i}\right)$ is a representation of the possibility of membership, and the degree of belongingness of $R_{\mathrm{wr}}\left(a_{i}\right)\in F\left(e_{i}\right)$ is indicated for each parameter $e_{i}$ by $F_{\mu }\left(e_{i}\right)=\left(F\left(e_{i}\right),\mu \left(e_{i}\right)\right).$

Example 1. Consider $C\subseteq F_{2}^{4}$ parameter set *E*, and define a fuzzy set on this parameter set *μ*( *e*) ∈ [0,1] for any set of *R*_*wr*_( *a*). And let

$$R_{\mathrm{wr}}\left(a\right)=\{R_{\mathrm{wr}}\left(0100\right),R_{\mathrm{wr}}\left(1010\right),R_{\mathrm{wr}}\left(1011\right),R_{\mathrm{wr}}\left(1110\right),R_{\mathrm{wr}}\left(0001\right),R_{\mathrm{wr}}\left(0111\right),R_{\mathrm{wr}}\left(1100\right),R_{\mathrm{wr}}\left(1111\right)\}$$

={0.2,0.5,0.8,0.6,0.4,0.9,0.3,1} be a fuzzy code of the eight computers under consideration.

Let $E=\left\{e_{1},e_{2},e_{3},e_{4}\right\}$ be the set of qualities of the given computers, where the symbols $e_{1}$ denote a super computer, $e_{2}$ a micro computer, $e_{3}$ a workstation computer, and $e_{4}$ a personal computer.

Let μ:E→[0,1] be a fuzzy code subset of *R*_*wr*_ : *C* → [0,1] defined as follows:

$$\mu \left(e_{1}\right)=R_{\mathrm{wr}}\;\left(0110\right)=0.5;$$

$$\mu \left(e_{2}\right)=R_{\mathrm{wr}}\left(1001\right)=0.6;$$

$$\mu \left(e_{3}\right)=R_{\mathrm{wr}}\left(1100\right)=0.3;\text{and}\;\mu \left(e_{4}\right)=R_{\mathrm{wr}}\left(1011\right)=0.8;$$

And we define a function

$\mathrm{F\mu}:E\to P\left(R_{\mathrm{wr}}\left(a_{i}\right)\right)\times R_{\mathrm{wr}}\left(a_{i}\right)$ , be defined as follows:

$$F_{\mu }\left(e_{1}\right)=\left(\{\frac{R_{\mathrm{wr}}\left(0001\right)}{0.4},\frac{R_{\mathrm{wr}}\left(0111\right)}{0.9},\frac{R_{\mathrm{wr}}\left(1110\right)}{0.6},\frac{R_{\mathrm{wr}}\left(1010\right)}{0.4}\},0.5\;\right)$$

$$F_{\mu }\left(e_{2}\right)=\left(\{\frac{R_{\mathrm{wr}}\left(1011\right)}{0.8},\frac{R_{\mathrm{wr}}\left(1110\right)}{0.6},\frac{R_{\mathrm{wr}}\left(1010\right)}{0.4},\frac{R_{\mathrm{wr}}\left(1111\right)}{1}\},0.6\;\right)$$

$$F_{\mu }\left(e_{3}\right)=\left(\{\frac{R_{\mathrm{wr}}\left(1110\right)}{0.6},\frac{R_{\mathrm{wr}}\left(0001\right)}{0.4},\frac{R_{\mathrm{wr}}\left(1011\right)}{0.8},\frac{R_{\mathrm{wr}}\left(0111\right)}{0.9}\},0.3\;\right)$$

$$F_{\mu }\left(e_{4}\right)=\left(\{\frac{R_{\mathrm{wr}}\left(0101\right)}{0.6},\frac{R_{\mathrm{wr}}\left(1100\right)}{0.3},\frac{R_{\mathrm{wr}}\left(1010\right)}{0.4},\frac{R_{\mathrm{wr}}\left(1111\right)}{1}\},0.8\;\right)$$

A Generalised fuzzy soft code over $\left(R_{\mathrm{wr}}\left(a\right),E\right)$ is then $F_{\mu }$ .

This can be represented in matrix form as follows:

$$F_{\mu }=\left[\begin{array}{cccc} 0.4 & 0.9 & 0.6 & 0.4|0.5\\ 0.8 & 0.6 & 0.4 & 1|0.6\\ 0.6 & 0.4 & 0.8 & 0.9|0.3\\ 0.6 & 0.3 & 0.4 & 1|0.8 \end{array}\right]$$

where the last column represents the values of $\mu \left(e_{i}\right),$ the row vectors $i^{\mathrm{th}}$ represent $F_{\mu }\left(e_{i}\right),$ and the column vector $\;i^{\mathrm{th}}$ represents $\;R_{\mathrm{wr}}\left(a_{i}\right)$ for some $a_{i}\in F_{2}^{4}$ . This will be referred to as the membership matrix of $F_{\mu }$ .

The GFSC is then can be defined as

$F_{\mu }\left(e_{i}\right),\left(e_{i}\right)=\left(F\left(e_{i}\right),\mu \left(e_{i}\right)\right),\forall e\in E,F\left(e\right)\in P\left(R_{\mathrm{wr}}\left(a_{i}\right)\right)$ and ∀i .

Definition 17. Given two GFSCs over $\left(R_{\mathrm{wr}}\left(a\right),E\right),$ let $F_{\mu }$ and $G_{\sigma }$ . Specifically, $F_{\mu }$ is a subset of $G_{\sigma }$ that is considered a generalised fuzzy soft code if

i
μ⊆σ

ii
F(e)⊆G(e),∀e∈E

Here, we compose $F_{\mu }\subseteq G_{\sigma }.$

Example 2. The GFSC $F_{\mu }$ over $\left(R_{\mathrm{wr}}\left(a\right),E\right)$ is the one presented in example 1. Let $G_{\sigma }$ be an additional GFSC defined as follows over $\left(R_{\mathrm{wr}}\left(a\right),E\right).$

$$G_{\sigma }\left(e_{1}\right)=\left(\{\frac{R_{\mathrm{wr}}\left(1001\right)}{0.5},\frac{R_{\mathrm{wr}}\left(0111\right)}{0.9},\frac{R_{\mathrm{wr}}\left(1011\right)}{0.8},\frac{R_{\mathrm{wr}}\left(0110\right)}{0.5}\},0.6\;\right)$$

$$G_{\sigma }\left(e_{2}\right)=\left(\{\frac{R_{\mathrm{wr}}\left(1011\right)}{0.8},\frac{R_{\mathrm{wr}}\left(0110\right)}{0.5},\frac{R_{\mathrm{wr}}\left(1110\right)}{0.6},\frac{R_{\mathrm{wr}}\left(1111\right)}{1}\},0.8\;\right)$$

$$G_{\sigma }\left(e_{3}\right)=\left(\{\frac{R_{\mathrm{wr}}\left(1011\right)}{0.8},\frac{R_{\mathrm{wr}}\left(1010\right)}{0.4},\frac{R_{\mathrm{wr}}\left(0111\right)}{0.9},\frac{R_{\mathrm{wr}}\left(1111\right)}{1}\},0.5\;\right)$$

$$G_{\sigma }\left(e_{4}\right)=\left(\{\frac{R_{\mathrm{wr}}\left(1011\right)}{0.8},\frac{R_{\mathrm{wr}}\left(1010\right)}{0.4},\frac{R_{\mathrm{wr}}\left(1001\right)}{0.5},\frac{R_{\mathrm{wr}}\left(1111\right)}{1}\},0.7\right)$$

Here σ and $R_{\mathrm{wr}}\left(a\right)$ are the respective mapping given by

σ:E→[0,1],

and

$$R_{\mathrm{wr}}\left(a\right):C\to 0,1]$$

Then, a generalised fuzzy soft code subset of $G_{\sigma }$ is $F_{\mu }$ .

Matrix representation of $G_{\sigma }$ is given by

$$G_{\sigma }=\left[\begin{array}{cccc} 0.5 & 0.9 & 0.8 & 0.9|0.6\\ 0.8 & 0.7 & 0.6 & 1|0.8\\ 0.8 & 0.4 & 0.9 & 1|0.5\\ 0.8 & 0.4 & 0.5 & 1|0.7 \end{array}\right]$$

Definition 18. (Intersection of GFSC). $F_{\mu }⋂G_{\sigma }$ represents the GFSC intersection of two GFSCs of $F_{\mu }$ and $G_{\sigma }$ over $\left(R_{\mathrm{wr}}\left(a\right),E\right)$ , and is defined as

$$\mathrm{F\mu}⋂G_{\sigma }=\min\left\{(F_{\mu }\left(e\right),\mu \left(e\right)),(G_{\sigma }\left(e\right),\sigma \left(e\right)),\forall e\in E\right\}.$$ (14)

Definition 19. (Union of GFSC) $F_{\mu }⋃G_{\sigma }$ represents the GFSC union of two GFSCs of $F_{\mu }$ and $G_{\sigma }$ over a soft universe $\left(R_{\mathrm{wr}}\left(a\right),E\right)$ , and is defined as

$$\mathrm{F\mu}⋃G_{\sigma }=\max\left\{(F_{\mu }\left(e\right),\mu \left(e\right)),(G_{\sigma }\left(e\right),\sigma \left(e\right)),\forall e\in E\right\}.$$ (15)

Definition 20. (Complement of GFSC). The following notation represents the complement of the GFSC of $F_{\mu }:{\left(F_{\mu }\right)}^{∁}$ or Subtracting the GFSC of each member of $\left(F_{\mu }\left(e\right),\mu \left(e\right)\right)$ from 1 yield ${F^{∁}}_{\mu^{∁}}$ . That is,

$$\;{F^{∁}}_{\mu^{∁}}=(1-F_{\mu }\left(e\right),1-\mu \left(e\right)$$ (16)

Theorem 1.
*If* $F_{\mu }$ *and* $G_{\sigma }$ *represent any GFSC over a shared soft universe* $\left(R_{\mathrm{wr}}\left(a\right),E\right)$ *, then the following result is hold:*

(i)
$F_{\mu }=F_{\mu }⋂F_{\mu }$

(ii)
$F_{\mu }=F_{\mu }⋃F_{\mu }$

(iii)
${\left({F^{∁}}_{\mu^{∁}}\right)}^{∁}=F_{\mu }$

(i)
${\left(F_{\mu }⋂G_{\sigma }\right)}^{∁}={F^{∁}}_{\mu^{∁}}⋃{G^{∁}}_{\mu^{∁}}$

(ii)
${\left(F_{\mu }⋃G_{\sigma }\right)}^{∁}={F^{∁}}_{\mu^{∁}}⋂{G^{∁}}_{\mu^{∁}}$

Proof:
The proof follows directly from the definition.

Theorem 2.
*The following properties are hold, if* (F,A) *and* (G,B) *are two generalised fuzzy soft codes over the common universe* $R_{\mathrm{wr}}\left(a\right).$

(i)
${\left(\left(F,A\right)⋃\left(G,B\right)\right)}^{∁}={\left(F,A\right)}^{∁}⋂{\left(G,B\right)}^{∁}$

(ii)
${\left(\left(F,A\right)⋂\left(G,B\right)\right)}^{∁}={\left(F,A\right)}^{∁}⋃{\left(G,B\right)}^{∁}$

Proof

(i)
${\left(\left(F,A\right)⋃\left(G,B\right)\right)}^{∁}=\left(F^{∁},\neg A\right)⋂\left(G^{∁},\neg B\right)=\left(H^{∁},\neg A\times \neg B\right),$

where $H\left(\neg e,\neg e^{\prime }\right)=F^{∁}\left(e\right)⋃G^{∁}\left(e^{\prime }\right)=\left(H,\neg \left(A\times B\right)\right))$

$$\Rightarrow {\left(F,A\right)}^{∁}⋃{\left(G,B\right)}^{∁}=\left(H,\neg \left(A\times B\right)\right)$$

Assume that, ((F,A)⋂(G,B))=(H,A×B))

$$\begin{array}{c} \Rightarrow {\left(\left(F,A\right)⋂\left(G,B\right)\right)}^{∁}={\left(H,A\times B\right)}^{∁}=\left(\mathrm{Hc},\neg \left(A\times B\right)\right),\forall \left(e,e^{\prime }\right)\in A\times B) \end{array}$$

$$\begin{array}{l} \Rightarrow H^{∁}\left(\neg e,\neg e^{\prime }\right)={\left(H\left(e,e^{\prime }\right)\right)}^{∁}\\ ={\left(F\left(e\right)⋂G\left(e^{\prime }\right)\right)}^{∁}\\ ={\left(F\left(e\right)\right)}^{∁}⋃{\left(G\left(e^{\prime }\right)\right)}^{∁}=F^{∁}\left(\neg e\right)⋃\mathrm{Gc}\left(\neg e^{\prime }\right)=\left(H,\neg \left(A\times B\right)\right))\\ \Rightarrow {\left(\left(F,A\right)⋂\left(G,B\right)\right)}^{∁} \end{array}$$

Thus, from our discussion we get the result,

$${\left(\left(F,A\right)⋃\left(G,B\right)\right)}^{∁}={\left(F,A\right)}^{∁}⋂{\left(G,B\right)}^{∁}$$

The proof of (ii) is similar to the above.

Definition 21. A generalised null fuzzy soft code, represented by $F_{\mu },$ is considered to be a generalised null fuzzy soft code if

$F_{\mu }:E\to P\left(R_{\mathrm{wr}}\left(a\right)\right)\times R_{\mathrm{wr}}\left(a\right)$ and $F_{\mu }\left(e\right)=\left(F\left(e\right),\mu \left(e\right)\right),$ where $F\left(e\right)=\bar{\;0}$ and

μ(e)=0,∀e∈E.

Definition 22. A GFSC is said to be a generalised absolute fuzzy soft code, denoted by $G_{\sigma }$ if $G_{\sigma }:E\to P\left(R_{\mathrm{wr}}\left(a\right)\right)\times R_{\mathrm{wr}}\left(a\right),$ where $G_{\sigma }\left(e\right)=\left(G\left(e\right),\sigma \left(e\right)\right)$ is defined by $G\left(e\right)=\bar{1},\forall e\in E,$ and σ(e)=1∀e∈E.

Theorem 3.
*Given any GFSC over* $\left(R_{\mathrm{wr}}\left(a\right),E\right)$ *, let’s say* $F_{\mu }$ *. The generalised null fuzzy soft code and generalised absolute fuzzy soft code over* $\left(R_{\mathrm{wr}}\left(a\right),E\right)$ *denoted as* $G_{\sigma }$ *and* $H_{\theta }$ *, respectively. Then, the following result is hold:*

(i)
$F_{\mu }⋂G_{\sigma }=G_{\sigma }$

(ii)
$F_{\mu }⋂H_{\theta }=F_{\mu }$

(iii)
$F_{\mu }⋃G_{\sigma }=F_{\mu }$

(iv)
$F_{\mu }⋃H_{\theta }=H_{\theta }$

(v)
$G_{\sigma }⋃H_{\theta }=H_{\theta }$

(vi)
$G_{\sigma }⋂H_{\theta }=G_{\sigma }$

Proof (i) Let $F_{\mu }$ be any GFSC over $\left(R_{\mathrm{wr}}\left(a\right),E\right)$ and let $G_{\sigma }$ be the generalised null fuzzy soft code over $\left(R_{\mathrm{wr}}\left(a\right),E\right)$ . Then

$$\begin{array}{c} F_{\mu }⋂G_{\sigma }=F_{\mu }\left(e\right)⋂G_{\sigma }\left(e\right)=\left(F\left(e\right),\mu \left(e\right)\right)⋂\left(G\left(e\right),\sigma \left(e\right)\right)\\ =\left(F\left(e\right),\mu \left(e\right)\right)⋂\left(\bar{0},0\right)=\left(\bar{0},0\right)=G_{\sigma } \end{array}$$

(ii-v) Similar to the proof of (i)

Definition 23. Given a universal set $U=R_{\mathrm{wr}}\left(a\right),$ consider two generalised fuzzy soft codes over $R_{\mathrm{wr}}\left(a\right):\left(F,A\right)$ and (G,B) . Then, the addition operation modulo between (F,A) and (G,B) is represented by (F,A)⊕(G,B), and the multiplication operation modulo between (F,A) and (G,B) is represented by (F,A)⊙(G,B) . The interpretation of these notations are as follows:

(i) ( F,A)⊕(G,B)=(H,a×b), where
(H,a×b)=F(a)⋃G(b)
(ii)
(F,A)⊙(G,B)=(H,a×b) , where
(H,a×b)=F(a)⋂G(b)anda×b∈A×B.

## 4. Relation on generalised fuzzy soft code and its application

In this subsection, we introduce generalised fuzzy soft codes and its properties.

Definition 24. Let $U=R_{\mathrm{wr}}\left(a_{i}\right)$ for which C is the universal element, consider the set of parameters be E, and $a_{i}$ is an element of a code vector. The fuzzy soft code universe is a pair (F,E).

Let $F:E\to P\left(R_{\mathrm{wr}}\left(a_{i}\right)\right),$ where all fuzzy code subsets of $R_{\mathrm{wr}}\left(a_{i}\right)\in \left[0,1\right]$ are collected as $P\left(R_{\mathrm{wr}}\left(a_{i}\right)\right)$ . And consider a mapping μ and $F_{\mu }$ such that

$$\mu :E\to R_{\mathrm{wr}}\left(a_{i}\right)$$ (12)

is a fuzzy subset of E and

$$\;F_{\mu }:E\to P\left(R_{\mathrm{wr}}\left(a_{i}\right)\right)\times R_{\mathrm{wr}}\left(a_{i}\right)$$ (13)

defined as $F_{\mu }\left(e_{i}\right)=\left(F\left(e_{i}\right),\mu \left(e_{i}\right)\right),\forall e\in E$ and $F\left(e_{i}\right)\in P\left(R_{\mathrm{wr}}\left(a_{i}\right)\right)$ . Then, $F_{\mu }$ is called a generalised fuzzy soft code (GFSC) over the soft universe.

$\left(R_{\mathrm{wr}}\left(a_{i}\right),E\right).\mu \left(e_{i}\right)$ is a representation of the possibility of membership, and the degree of belongingness of $R_{\mathrm{wr}}\left(a_{i}\right)\in F\left(e_{i}\right)$ is indicated for each parameter $e_{i}$ by $F_{\mu }\left(e_{i}\right)=\left(F\left(e_{i}\right),\mu \left(e_{i}\right)\right).$

Example 3. Consider $C\subseteq F_{2}^{4}$ parameter set *E*, and define a fuzzy set on this parameter set *μ*( *e*) ∈ [0,1] for any set of *R*_*wr*_( *a*). And let

$$R_{\mathrm{wr}}\left(a\right)=\{R_{\mathrm{wr}}\left(0100\right),R_{\mathrm{wr}}\left(1010\right),R_{\mathrm{wr}}\left(1011\right),R_{\mathrm{wr}}\left(1110\right),R_{\mathrm{wr}}\left(0001\right),R_{\mathrm{wr}}\left(0111\right),R_{\mathrm{wr}}\left(1100\right),R_{\mathrm{wr}}\left(1111\right)\}$$

={0.2,0.5,0.8,0.6,0.4,0.9,0.3,1} be a fuzzy code of the eight computers under consideration.

Let $E=\left\{e_{1},e_{2},e_{3},e_{4}\right\}$ be the set of qualities of the given computers, where the symbols $e_{1}$ denote a super computer, $e_{2}$ a micro computer, $e_{3}$ a workstation computer, and $e_{4}$ a personal computer.

Let μ:E→[0,1] be a fuzzy code subset of *R*_*wr*_ : *C* → [0,1] defined as follows:

$$\mu \left(e_{1}\right)=R_{\mathrm{wr}}\;\left(0110\right)=0.5;$$

$$\mu \left(e_{2}\right)=R_{\mathrm{wr}}\left(1001\right)=0.6;$$

$$\mu \left(e_{3}\right)=R_{\mathrm{wr}}\left(1100\right)=0.3;\text{and}\;\mu \left(e_{4}\right)=R_{\mathrm{wr}}\left(1011\right)=0.8;$$

And we define a function

$\mathrm{F\mu}:E\to P\left(R_{\mathrm{wr}}\left(a_{i}\right)\right)\times R_{\mathrm{wr}}\left(a_{i}\right)$ , be defined as follows:

$$F_{\mu }\left(e_{1}\right)=\left(\{\frac{R_{\mathrm{wr}}\left(0001\right)}{0.4},\frac{R_{\mathrm{wr}}\left(0111\right)}{0.9},\frac{R_{\mathrm{wr}}\left(1110\right)}{0.6},\frac{R_{\mathrm{wr}}\left(1010\right)}{0.4}\},0.5\;\right)$$

$$F_{\mu }\left(e_{2}\right)=\left(\{\frac{R_{\mathrm{wr}}\left(1011\right)}{0.8},\frac{R_{\mathrm{wr}}\left(1110\right)}{0.6},\frac{R_{\mathrm{wr}}\left(1010\right)}{0.4},\frac{R_{\mathrm{wr}}\left(1111\right)}{1}\},0.6\;\right)$$

$$F_{\mu }\left(e_{3}\right)=\left(\{\frac{R_{\mathrm{wr}}\left(1110\right)}{0.6},\frac{R_{\mathrm{wr}}\left(0001\right)}{0.4},\frac{R_{\mathrm{wr}}\left(1011\right)}{0.8},\frac{R_{\mathrm{wr}}\left(0111\right)}{0.9}\},0.3\;\right)$$

$$F_{\mu }\left(e_{4}\right)=\left(\{\frac{R_{\mathrm{wr}}\left(0101\right)}{0.6},\frac{R_{\mathrm{wr}}\left(1100\right)}{0.3},\frac{R_{\mathrm{wr}}\left(1010\right)}{0.4},\frac{R_{\mathrm{wr}}\left(1111\right)}{1}\},0.8\;\right)$$

A Generalised fuzzy soft code over $\left(R_{\mathrm{wr}}\left(a\right),E\right)$ is then $F_{\mu }$ .

This can be represented in matrix form as follows:

$$F_{\mu }=\left[\begin{array}{cccc} 0.4 & 0.9 & 0.6 & 0.4|0.5\\ 0.8 & 0.6 & 0.4 & 1|0.6\\ 0.6 & 0.4 & 0.8 & 0.9|0.3\\ 0.6 & 0.3 & 0.4 & 1|0.8 \end{array}\right]$$

where the last column represents the values of $\mu \left(e_{i}\right),$ the row vectors $i^{\mathrm{th}}$ represent $F_{\mu }\left(e_{i}\right),$ and the column vector $\;i^{\mathrm{th}}$ represents $\;R_{\mathrm{wr}}\left(a_{i}\right)$ for some $a_{i}\in F_{2}^{4}$ . This will be referred to as the membership matrix of $F_{\mu }$ .

The GFSC is then can be defined as

$F_{\mu }\left(e_{i}\right),\left(e_{i}\right)=\left(F\left(e_{i}\right),\mu \left(e_{i}\right)\right),\forall e\in E,F\left(e\right)\in P\left(R_{\mathrm{wr}}\left(a_{i}\right)\right)$ and ∀i .

Definition 25. Given two GFSCs over $\left(R_{\mathrm{wr}}\left(a\right),E\right),$ let $F_{\mu }$ and $G_{\sigma }$ . Specifically, $F_{\mu }$ is a subset of $G_{\sigma }$ that is considered a generalised fuzzy soft code if

i
μ⊆σ

ii
F(e)⊆G(e),∀e∈E

Here, we compose $F_{\mu }\subseteq G_{\sigma }.$

Example 4. The GFSC $F_{\mu }$ over $\left(R_{\mathrm{wr}}\left(a\right),E\right)$ is the one presented in example 1. Let $G_{\sigma }$ be an additional GFSC defined as follows over $\left(R_{\mathrm{wr}}\left(a\right),E\right).$

$$G_{\sigma }\left(e_{1}\right)=\left(\{\frac{R_{\mathrm{wr}}\left(1001\right)}{0.5},\frac{R_{\mathrm{wr}}\left(0111\right)}{0.9},\frac{R_{\mathrm{wr}}\left(1011\right)}{0.8},\frac{R_{\mathrm{wr}}\left(0110\right)}{0.5}\},0.6\;\right)$$

$$G_{\sigma }\left(e_{2}\right)=\left(\{\frac{R_{\mathrm{wr}}\left(1011\right)}{0.8},\frac{R_{\mathrm{wr}}\left(0110\right)}{0.5},\frac{R_{\mathrm{wr}}\left(1110\right)}{0.6},\frac{R_{\mathrm{wr}}\left(1111\right)}{1}\},0.8\;\right)$$

$$G_{\sigma }\left(e_{3}\right)=\left(\{\frac{R_{\mathrm{wr}}\left(1011\right)}{0.8},\frac{R_{\mathrm{wr}}\left(1010\right)}{0.4},\frac{R_{\mathrm{wr}}\left(0111\right)}{0.9},\frac{R_{\mathrm{wr}}\left(1111\right)}{1}\},0.5\;\right)$$

$$G_{\sigma }\left(e_{4}\right)=\left(\{\frac{R_{\mathrm{wr}}\left(1011\right)}{0.8},\frac{R_{\mathrm{wr}}\left(1010\right)}{0.4},\frac{R_{\mathrm{wr}}\left(1001\right)}{0.5},\frac{R_{\mathrm{wr}}\left(1111\right)}{1}\},0.7\right)$$

Here σ and $R_{\mathrm{wr}}\left(a\right)$ are the respective mapping given by

σ:E→[0,1],

and

$$R_{\mathrm{wr}}\left(a\right):C\to 0,1]$$

Then, a generalised fuzzy soft code subset of $G_{\sigma }$ is $F_{\mu }$ .

Matrix representation of $G_{\sigma }$ is given by

$$G_{\sigma }=\left[\begin{array}{cccc} 0.5 & 0.9 & 0.8 & 0.9|0.6\\ 0.8 & 0.7 & 0.6 & 1|0.8\\ 0.8 & 0.4 & 0.9 & 1|0.5\\ 0.8 & 0.4 & 0.5 & 1|0.7 \end{array}\right]$$

Definition 26. (Intersection of GFSC). $F_{\mu }⋂G_{\sigma }$ represents the GFSC intersection of two GFSCs of $F_{\mu }$ and $G_{\sigma }$ over $\left(R_{\mathrm{wr}}\left(a\right),E\right)$ , and is defined as

$$\mathrm{F\mu}⋂G_{\sigma }=\min\left\{(F_{\mu }\left(e\right),\mu \left(e\right)),(G_{\sigma }\left(e\right),\sigma \left(e\right)),\forall e\in E\right\}.$$ (14)

Definition 27. (Union of GFSC) $F_{\mu }⋃G_{\sigma }$ represents the GFSC union of two GFSCs of $F_{\mu }$ and $G_{\sigma }$ over a soft universe $\left(R_{\mathrm{wr}}\left(a\right),E\right)$ , and is defined as

$$\mathrm{F\mu}⋃G_{\sigma }=\max\left\{(F_{\mu }\left(e\right),\mu \left(e\right)),(G_{\sigma }\left(e\right),\sigma \left(e\right)),\forall e\in E\right\}.$$ (15)

Definition 28. (Complement of GFSC). The following notation represents the complement of the GFSC of $F_{\mu }:{\left(F_{\mu }\right)}^{∁}$ or Subtracting the GFSC of each member of $\left(F_{\mu }\left(e\right),\mu \left(e\right)\right)$ from 1 yield ${F^{∁}}_{\mu^{∁}}$ . That is,

$$\;{F^{∁}}_{\mu^{∁}}=(1-F_{\mu }\left(e\right),1-\mu \left(e\right)$$ (16)

Theorem 1.
*If* $F_{\mu }$ *and* $G_{\sigma }$ *represent any GFSC over a shared soft universe* $\left(R_{\mathrm{wr}}\left(a\right),E\right)$ *, then the following result is hold:*

(i)
$F_{\mu }=F_{\mu }⋂F_{\mu }$

(ii)
$F_{\mu }=F_{\mu }⋃F_{\mu }$

(iii)
${\left({F^{∁}}_{\mu^{∁}}\right)}^{∁}=F_{\mu }$

(i)
${\left(F_{\mu }⋂G_{\sigma }\right)}^{∁}={F^{∁}}_{\mu^{∁}}⋃{G^{∁}}_{\mu^{∁}}$

(ii)
${\left(F_{\mu }⋃G_{\sigma }\right)}^{∁}={F^{∁}}_{\mu^{∁}}⋂{G^{∁}}_{\mu^{∁}}$

Proof:
The proof follows directly from the definition.

Theorem 2.
*The following properties are hold, if* (F,A) *and* (G,B) *are two generalised fuzzy soft codes over the common universe* $R_{\mathrm{wr}}\left(a\right).$

(i)
${\left(\left(F,A\right)⋃\left(G,B\right)\right)}^{∁}={\left(F,A\right)}^{∁}⋂{\left(G,B\right)}^{∁}$

(ii)
${\left(\left(F,A\right)⋂\left(G,B\right)\right)}^{∁}={\left(F,A\right)}^{∁}⋃{\left(G,B\right)}^{∁}$

Proof

(i)
${\left(\left(F,A\right)⋃\left(G,B\right)\right)}^{∁}=\left(F^{∁},\neg A\right)⋂\left(G^{∁},\neg B\right)=\left(H^{∁},\neg A\times \neg B\right),$

where $H\left(\neg e,\neg e^{\prime }\right)=F^{∁}\left(e\right)⋃G^{∁}\left(e^{\prime }\right)=\left(H,\neg \left(A\times B\right)\right))$

$$\Rightarrow {\left(F,A\right)}^{∁}⋃{\left(G,B\right)}^{∁}=\left(H,\neg \left(A\times B\right)\right)$$

Assume that, ((F,A)⋂(G,B))=(H,A×B))

$$\begin{array}{c} \Rightarrow {\left(\left(F,A\right)⋂\left(G,B\right)\right)}^{∁}={\left(H,A\times B\right)}^{∁}=\left(\mathrm{Hc},\neg \left(A\times B\right)\right),\forall \left(e,e^{\prime }\right)\in A\times B) \end{array}$$

$$\begin{array}{l} \Rightarrow H^{∁}\left(\neg e,\neg e^{\prime }\right)={\left(H\left(e,e^{\prime }\right)\right)}^{∁}\\ ={\left(F\left(e\right)⋂G\left(e^{\prime }\right)\right)}^{∁}\\ ={\left(F\left(e\right)\right)}^{∁}⋃{\left(G\left(e^{\prime }\right)\right)}^{∁}=F^{∁}\left(\neg e\right)⋃\mathrm{Gc}\left(\neg e^{\prime }\right)=\left(H,\neg \left(A\times B\right)\right))\\ \Rightarrow {\left(\left(F,A\right)⋂\left(G,B\right)\right)}^{∁} \end{array}$$

Thus, from our discussion we get the result,

$${\left(\left(F,A\right)⋃\left(G,B\right)\right)}^{∁}={\left(F,A\right)}^{∁}⋂{\left(G,B\right)}^{∁}$$

The proof of (ii) is similar to the above.

Definition 29. A generalised null fuzzy soft code, represented by $F_{\mu },$ is considered to be a generalised null fuzzy soft code if

$F_{\mu }:E\to P\left(R_{\mathrm{wr}}\left(a\right)\right)\times R_{\mathrm{wr}}\left(a\right)$ and $F_{\mu }\left(e\right)=\left(F\left(e\right),\mu \left(e\right)\right),$ where $F\left(e\right)=\bar{\;0}$ and

μ(e)=0,∀e∈E.

Definition 30. A GFSC is said to be a generalised absolute fuzzy soft code, denoted by $G_{\sigma }$ if $G_{\sigma }:E\to P\left(R_{\mathrm{wr}}\left(a\right)\right)\times R_{\mathrm{wr}}\left(a\right),$ where $G_{\sigma }\left(e\right)=\left(G\left(e\right),\sigma \left(e\right)\right)$ is defined by $G\left(e\right)=\bar{1},\forall e\in E,$ and σ(e)=1∀e∈E.

Theorem 3.
*Given any GFSC over* $\left(R_{\mathrm{wr}}\left(a\right),E\right)$ *, let’s say* $F_{\mu }$ *. The generalised null fuzzy soft code and generalised absolute fuzzy soft code over* $\left(R_{\mathrm{wr}}\left(a\right),E\right)$ *denoted as* $G_{\sigma }$ *and* $H_{\theta }$ *, respectively. Then, the following result is hold:*

(i)
$F_{\mu }⋂G_{\sigma }=G_{\sigma }$

(ii)
$F_{\mu }⋂H_{\theta }=F_{\mu }$

(iii)
$F_{\mu }⋃G_{\sigma }=F_{\mu }$

(iv)
$F_{\mu }⋃H_{\theta }=H_{\theta }$

(v)
$G_{\sigma }⋃H_{\theta }=H_{\theta }$

(vi)
$G_{\sigma }⋂H_{\theta }=G_{\sigma }$

Proof (i) Let $F_{\mu }$ be any GFSC over $\left(R_{\mathrm{wr}}\left(a\right),E\right)$ and let $G_{\sigma }$ be the generalised null fuzzy soft code over $\left(R_{\mathrm{wr}}\left(a\right),E\right)$ . Then

$$\begin{array}{c} F_{\mu }⋂G_{\sigma }=F_{\mu }\left(e\right)⋂G_{\sigma }\left(e\right)=\left(F\left(e\right),\mu \left(e\right)\right)⋂\left(G\left(e\right),\sigma \left(e\right)\right)\\ =\left(F\left(e\right),\mu \left(e\right)\right)⋂\left(\bar{0},0\right)=\left(\bar{0},0\right)=G_{\sigma } \end{array}$$

(ii-v) Similar to the proof of (i)

Definition 31. Given a universal set $U=R_{\mathrm{wr}}\left(a\right),$ consider two generalised fuzzy soft codes over $R_{\mathrm{wr}}\left(a\right):\left(F,A\right)$ and (G,B) . Then, the addition operation modulo between (F,A) and (G,B) is represented by (F,A)⊕(G,B), and the multiplication operation modulo between (F,A) and (G,B) is represented by (F,A)⊙(G,B) . The interpretation of these notations are as follows:

(i) ( F,A)⊕(G,B)=(H,a×b), where
(H,a×b)=F(a)⋃G(b)
(ii)
(F,A)⊙(G,B)=(H,a×b) , where
(H,a×b)=F(a)⋂G(b)anda×b∈A×B.

## Consent to participate and ethics approval

This research paper is an original work that has not been published or submitted elsewhere at the same time.

## Data Availability

No data are associated with this article.
