# Supplementary material for: New Approaches of Generalised Fuzzy Soft sets on fuzzy Codes and Its Properties on Decision-Makings
Source: F1000Res. 2025 Apr 9;13:1461. Originally published 2024 Dec 2. [Version 2] doi: 10.12688/f1000research.158747.2 (PMC12038337; doi:10.12688/f1000research.158747.2)
Supplement: Peer review history

# Reviewer report: Reviewer response for version 2

**DOI:** 10.5256/f1000research.179056.r377009

**Published:** 2025-04-28

**Authors:** Rana Muhammad Zulqarnain

**Affiliations:** 1 Zhejiang Normal University, Zhejiang, China

The authors revised the manuscript very well. I have no further questions for the authors.

Is the work clearly and accurately presented and does it cite the current literature?

Partly

If applicable, is the statistical analysis and its interpretation appropriate?

Not applicable

Are all the source data underlying the results available to ensure full reproducibility?

Yes

Is the study design appropriate and is the work technically sound?

Partly

Are the conclusions drawn adequately supported by the results?

Partly

Are sufficient details of methods and analysis provided to allow replication by others?

Yes

Reviewer Expertise:

Fuzzy extensions.

I confirm that I have read this submission and believe that I have an appropriate level of expertise to confirm that it is of an acceptable scientific standard.

# Reviewer report: Reviewer response for version 2

**DOI:** 10.5256/f1000research.179056.r377656

**Published:** 2025-04-22

**Authors:** Ajoy Kanti Das

**Affiliations:** 1 Tripura University, Suryamani Nagar, Tripura, India

**REVIEW REPORT**

**New Approaches of Generalised Fuzzy Soft sets on fuzzy Codes and Its Properties on Decision-Makings**

 This manuscript proposes a new concept called Generalised Fuzzy Soft Code (GFSC), integrating the ideas of generalised fuzzy soft sets with fuzzy codes. The paper attempts to formalize definitions, operations (union, intersection, complement), and matrix representations of GFSC, with illustrative examples and basic theorems. The paper is well-structured, with clear objectives and systematic development of theoretical models. However, there are several areas where the manuscript could benefit from improvement, both in terms of structure and content. Some comments are suggested to improve the quality of this paper.

**Suggestions for Improvement:**

1. Check and correct all typographical and grammatical errors. For example: (i) *“on fuzzy Codes and **Its** Properties...” in the Title should be: “on Fuzzy Codes and **Their** Properties...”*
2. In the third paragraph of the Introduction, the phrase “formationed works” is used, which is not a valid expression. This should be replaced with an appropriate and grammatically correct alternative, such as “foundational works” or “previous studies”, depending on the intended meaning.
3. Abbreviations should be defined before their first use. This is not consistently followed in the manuscript. For example, IFSS appears in the Introduction but is not explained. It is recommended to define all abbreviations upon first use and include a list of abbreviations in a table after the abstract or before Section 1.
4. The citation for Definition 6 is incorrect. It is currently cited as (Molodtsov 2011), but it should be (Molodtsov, 1999), as the original soft set theory was introduced in that year. Please update this citation accordingly.
5. In **Definition 9**, the phrase *“The universal set of elements is = {x₁,x₂,…,x* *ₙ}* *”* is incorrect and incomplete. It should be written as: **“ *The universal set of elements is*** U= *{x₁,x₂,…,x* *ₙ}* *”*
6. Set and function symbols are sometimes misused or introduced without adequate explanation. For example, in Definition 16, the statement “Let U = Rwr(ai) for which C is the universal element” is unclear, what and represent in this context. Please revise this definition to clearly specify the meaning and roles of all symbols and sets involved.
7. I strongly recommend double-checking all the symbols and formulas to ensure accuracy in indices, notation, and conceptual consistency. To improve readability, consider adding a table summarizing all mathematical symbols and their definitions used in the manuscript. This addition would enhance clarity and accessibility, especially for readers who may not be familiar with the specific notation employed.
8. While the theoretical contributions are clear, the paper would benefit from a stronger justification of why Generalised Fuzzy Soft Sets on Fuzzy Codes are useful or necessary. Specifically, the manuscript lacks discussion on real-world problems or domains where these models offer distinct advantages over classical or existing fuzzy set approaches. It is recommended to add a dedicated section that illustrates practical scenarios—such as in medical diagnosis, information coding, or decision-making under uncertainty—where Generalised Fuzzy Soft Sets on Fuzzy Codes provide improved interpretability, flexibility, or accuracy. This would significantly enhance the paper’s relevance and motivate the proposed theoretical framework.
9. The focus is primarily theoretical; real-world applications or potential use cases are not discussed. Expand the literature review by including more recent research on fuzzy soft set and its applications in various domains. I suggest: Refer: 1,2,3,4,5,6. 
10. Ensure consistent formatting of equations, as some appear cramped or unclear.
11. Include a future research section that outlines the next steps for further research to emphasize the study’s broader relevance.
12. Please recheck all references to ensure they are written correctly and include complete information. Some references are incomplete. For instance, the reference to Muhammad et al. (2023) is incomplete — the journal name, volume number, and page range are missing and should be included.

The paper makes a significant theoretical contribution and has strong potential for real-world applications but requires significant revision in terms of language, structure, and typesetting before it can be accepted for indexing. **Recommendation**: Accept with revisions.

Is the work clearly and accurately presented and does it cite the current literature?

Partly

If applicable, is the statistical analysis and its interpretation appropriate?

Yes

Are all the source data underlying the results available to ensure full reproducibility?

No source data required

Is the study design appropriate and is the work technically sound?

Partly

Are the conclusions drawn adequately supported by the results?

Yes

Are sufficient details of methods and analysis provided to allow replication by others?

Yes

Reviewer Expertise:

Fuzzy set theory and applications, soft set theory, Fuzzy extensions, Sequence spaces, Water quality assessment

I confirm that I have read this submission and believe that I have an appropriate level of expertise to confirm that it is of an acceptable scientific standard, however I have significant reservations, as outlined above.

## References

[1] : A New Kind of Generalized Pythagorean Fuzzy Soft Set and Its Application in Decision-Making. *Computer Modeling in Engineering & Sciences* .2023;136(3) : 10.32604/cmes.2023.026021 2861-2871 10.32604/cmes.2023.026021

[2] : A new type of generalized picture fuzzy soft set and its application in decision making. *Journal of Intelligent & Fuzzy Systems* .2021;40(6) : 10.3233/JIFS-201706 12459-12475 10.3233/JIFS-201706

[3] : An Advanced Approach to Fuzzy Soft Group Decision-Making Using Weighted Average Ratings. *SN Computer Science* .2021;2(6) : 10.1007/s42979-021-00873-5 10.1007/s42979-021-00873-5

[4] : Einstein-operations on fuzzy soft multi sets and decision making. *Boletim da Sociedade Paranaense de Matemática* .2022;40: 10.5269/bspm.32546 1-10 10.5269/bspm.32546

[5] : FP-intuitionistic multi fuzzy N-soft set and its induced FP-Hesitant N soft set in decision-making. *Decision Making: Applications in Management and Engineering* .2022;5(1) : 10.31181/dmame181221045d 67-89 10.31181/dmame181221045d

[6] : New generalization of fuzzy soft sets: $ (a, b) $-Fuzzy soft sets. *AIMS Mathematics* .2023;8(2) : 10.3934/math.2023155 2995-3025 10.3934/math.2023155

# Reviewer report: Reviewer response for version 1

**DOI:** 10.5256/f1000research.174381.r367664

**Published:** 2025-03-11

**Authors:** Rana Muhammad Zulqarnain

**Affiliations:** 1 Zhejiang Normal University, Zhejiang, China

1. I encourage you to improve the abstract section precisely with your core contributions.
2. Keywords are inappropriate; please add proper keywords for readers' convenience.
3. Considering the innovative nature of this methodology and its potential influence on academic research and industrial practices, the authors should emphasize the interdisciplinary aspects of their study, particularly regarding evolutionary economics and organizational behavior.
4. The problem statement and motivation is given. Please explain the problem in detail.
5. There are no research questions, so please add the research question for the reader's convenience.
6. The introduction and theoretical background are not enough. I suggest some important studies for authors to improve the introduction and background of this manuscript. Also, I strongly recommend reading these studies carefully and cite in the introduction and background sections. The literature review would not have been acceptable to me without the following studies in the introduction and references section. So, carefully revised the introduction and background. (a): Some Einstein geometric aggregation operators for Q-rung orthopair fuzzy soft set with their application in MCDM. (b): Aggregation Operators for Interval-Valued Pythagorean Fuzzy Soft Set with Their Application to Solve Multi-Attribute Group Decision Making Problem. (c): Einstein Aggregation Operators for Pythagorean Fuzzy Soft Sets with Their Application in Multiattribute Group Decision‐Making. (d): An interaction and feedback mechanism-based group decision-making for emergency medical supplies supplier selection using T-spherical fuzzy information. (d): Optimal cloud service provider selection: An MADM framework on correlation-based TOPSIS with interval-valued q-rung orthopair fuzzy soft set. (e): Algorithms for a Generalized Multipolar Neutrosophic Soft Set with Information Measures to Solve Medical Diagnoses and Decision‐Making Problems. (f): Extension of Einstein average aggregation operators to medical diagnostic approach under Q-rung orthopair fuzzy soft set. (g): Novel multicriteria decision making approach for interactive aggregation operators of q-rung orthopair fuzzy soft set. (h): Selection of unmanned aerial vehicles for precision agriculture using interval-valued q-rung orthopair fuzzy information based TOPSIS method. (i): An integrated group decision-making technique under interval-valued probabilistic linguistic T-spherical fuzzy information and its application to the selection of cloud storage provider. (j): Einstein ordered weighted aggregation operators for Pythagorean fuzzy hypersoft set with its application to solve MCDM problem. (k): Supplier Selection in Green Supply Chain Management using Correlation-based TOPSIS in a q-rung Orthopair Fuzzy Soft Environment. (l): An Intelligent MCGDM Model in Green Suppliers Selection Using Interactional Aggregation Operators for Interval-Valued Pythagorean Fuzzy Soft Sets. (m): Extension of correlation coefficient based TOPSIS technique for interval-valued Pythagorean fuzzy soft set: A case study in extract, transform, and load techniques. (n): Assessment of bio-medical waste disposal techniques using interval-valued q-rung orthopair fuzzy soft set based EDAS method. (o): Einstein Hybrid Structure of q-Rung Orthopair Fuzzy Soft Set and Its Application for Diagnosis of Waterborne Infectious Disease. (p): Transportation decisions in supply chain management using interval-valued q-rung orthopair fuzzy soft information.
7. Please clarify which of the assumptions is new in this area in the problem definition.
8. How did you consider the data in the case study?
9. The input section mentions constructing a group of decision-makers but doesn't clearly explain how they influence the process. Similarly, the output section could benefit from more detail on the final results, such as how the ranking is used for decision-making.
10. The authors should discuss how their presented work is needed at the time and whether it will be helpful to the scientific community.
11. The role of weights in the aggregation process could be explained more explicitly, particularly how they are assigned to each alternative and how they impact the final decision-making process.
12. How are the experts’ weights determined, and how do they influence the decision-making process?
13. Comparative analysis section in the manuscript is not enough, please add more comparative studies to prove the validity of the proposed model.
14. Can the research's shortcomings be commented on, particularly regarding the number of participants and the range of sectors addressed in the focus groups?
15. Please bring and focus on future research directions.

Is the work clearly and accurately presented and does it cite the current literature?

Partly

If applicable, is the statistical analysis and its interpretation appropriate?

Not applicable

Are all the source data underlying the results available to ensure full reproducibility?

Yes

Is the study design appropriate and is the work technically sound?

Partly

Are the conclusions drawn adequately supported by the results?

Partly

Are sufficient details of methods and analysis provided to allow replication by others?

Yes

Reviewer Expertise:

Fuzzy extensions.

I confirm that I have read this submission and believe that I have an appropriate level of expertise to confirm that it is of an acceptable scientific standard, however I have significant reservations, as outlined above.

# Response

**Published:** 2025-03-12

**Authors:** Masresha Wassie Woldie

**Affiliations:** Bahir Dar University Department of Mathematics, Bahir Dar, Amhara, Ethiopia

We incorporated all comments as the reviewer's suggestions.

# Reviewer report: Reviewer response for version 1

**DOI:** 10.5256/f1000research.174381.r367656

**Published:** 2025-03-05

**Authors:** Faruk Karaaslan

**Affiliations:** 1 Çankırı Karatekin University,, Çankırı, Turkey

**Review Report for “New Approaches of Generalised Fuzzy Soft sets on fuzzy**

**Codes and Its Properties on Decision-Makings”**

 A new aspect of this paper is to introduce and develop the definition and application of generalized fuzzy soft code by combining the concepts of fuzzy soft sets and fuzzy codes. The definition and features of relations on generalized fuzzy soft codes are proposed. But there are some problems in the paper. After the following revisions, Paper can be revaluated for indexing.

- In definition 4, *R* *wr* *c* *i* *∪*  *R* *wr* *c* *j* *=*max⁡ *(….)*   subindices should be checked and corrected.
- Citation of definition 6 should be corected, because soft set was defined by Molodtsov (1999).  Also, in same definition, “P(U) be represents…” should be replaced by “P(U) represents”. Definition 6, line 8-9, “ *e∈A.*   where… “ should be corrected.  Also, sentences beginning with “We might consider…” I think that soft set is a special type of Zadeh’s fuzz sets.  Check this sentence
- In definition 8,  should be replaced by ”
- In definition 9. “The universal set of elements is ,…”  should be rewritten as “The universal set of elements is ,…”  In same definition,  where ”  should be checked and corrected.
- In Definition 12,  “t-norm” should be replaced by “s-norm or t-conorm”
- In Definition 13, “  ”  should be replaced by “ ”  and, “*”  should be “t-norm”
- In definition 14, 15,  authors use some notations without they give an explanation, for example “ ”  and . They should be explained.
- In example 1.  I see that basic idea is that authors take the  parameter set E, and define a fuzzy set on this parameter set; also, they take  for any set of .
- In example 1,  “Let …”  What does it mean? According to definition of ,  Why you write ”  is not true expression as mathematically.
- In example 2,  “…in example 3.2 ”.  Where is the example 3.2?
- In example 2,  should be replaced by ”.
- In example 2,  “e generalized fuzzy soft code subset of *G* *σ*  is** .” This is not true, it should be checked.
- In definition 18, minimum operation is not defined between pairs  and   So if author write the . Same problem is available for Definition 19.
- We know that   is a fuzzy set. How can you maket he substruction operation between a number and fuzzy set.  Actually I understand what you make, bu this writting is not true mathematical. Check and  rewrite them.
- When I investigate the proof of theorem 2, I see that it is same with the proof for generalized fuzzy soft sets.  What is the difference between generalized fuzzy soft code and generalized fuzzy soft set. I think that Theorem 2, and 3 are trivial results of the generalized fuzzy soft set.
- In Definition 24, “GFSC”  should be replaced by “GFSCs”.
- The application part related to generalized fuzzy soft code relations is elementary and we can apply this to all generalized fuzzy soft sets. What is the importance of this application in terms of the codes? The results of the application are independent of codes. The importance and effect of the codes must be explained in the paper.
- The introduction part should be improved.
- The paper could benefit from refinement in terms of mathematical writing style to enhance clarity and precision.
- The title should be rewritten.

**Decision: Major Revision**

Is the work clearly and accurately presented and does it cite the current literature?

Partly

If applicable, is the statistical analysis and its interpretation appropriate?

I cannot comment. A qualified statistician is required.

Are all the source data underlying the results available to ensure full reproducibility?

Yes

Is the study design appropriate and is the work technically sound?

Partly

Are the conclusions drawn adequately supported by the results?

Partly

Are sufficient details of methods and analysis provided to allow replication by others?

Partly

Reviewer Expertise:

Fuzzy set, soft sets, decision-making, fuzzy graphs, fuzzy algebraic.

I confirm that I have read this submission and believe that I have an appropriate level of expertise to confirm that it is of an acceptable scientific standard, however I have significant reservations, as outlined above.

# Response

**Published:** 2025-03-06

**Authors:** Masresha Wassie Woldie

**Affiliations:** Bahir Dar University Department of Mathematics, Bahir Dar, Amhara, Ethiopia

1. we incorporated all suggested comments and submitted the corrected version.

 2. The difference between a generalised fuzzy soft set and generalised fuzzy soft code is that the generalised fuzzy soft set takes ''the set of fuzzy sets''. However, the generalised fuzzy soft code takes ''the set of fuzzy code sets''.

# Reviewer report: Reviewer response for version 1

**DOI:** 10.5256/f1000research.174381.r346175

**Published:** 2025-01-03

**Authors:** Tahir Mahmood

**Affiliations:** 1 International Islamic University Islamabad, Islamabad, Pakistan

The paper "New Approaches of Generalised Fuzzy Soft sets on fuzzy Codes and Its Properties on Decision-Makings " aims to develop a decision-making approach based on generalized fuzzy soft sets. The subject of the paper fits the aims and scope of the Journal. More detailed comments are as follows and I recommend the following major revision.

- Revise the title as “New Approaches of Generalized Fuzzy Soft Sets on Fuzzy Codes and Its Applications in Decision-Makings”
- The abstract is not written well. It has a lot of unexplained abbreviations. The authors didn’t adequately introduce the topic they are covering in the paper. It lacks an Information Classification: General adequate background of the problem highlighting the motivation for the study, main results, and conclusions. Further it is too short.
- Keywords are not enough to understand the theme of the manuscript. These should be enhanced.
- Most of what is currently Introduction should be moved from another section or should be extended, since it is too short to be useful and it should deal with the background of the problem and literature review effectively. Within this section, the authors should highlight the research gaps that this paper is trying to cover. The authors should discuss in the introduction a short overview of the problem and highlight the purpose and the aim of the study, as well as the main results, conclusions, and scientific contributions. It also lacks the historical background and literature review of the proposed study including the framework of soft set.
- The paper does not have a proper discussion. The authors did not discuss how the results can be interpreted from the perspective of previous studies. Discussion should clearly and concisely explain the significance of the obtained results to demonstrate the actual contribution of the article to this field of research when compared with the existing and studied literature.
- In the manuscript all the references should be cited correctly. For Example, in Definition 6, the reference (Kong, Wang, and Wu 2011) is cited, which needs correction.
- In subsection 3.1 and hence in the whole manuscript “generalised” should be “generalized”.
- The authors didn’t explain the limitations and implications of the proposed approach.
- Applications of the proposed theory needs attention as it is not properly treated.
- The conclusion is scarce. The authors should address the main research questions and highlight the main results, conclusions, contributions, and future research directions in the conclusion. Future scope of research should be multidirectional like bipolar soft sets, double framed soft sets, soft algebraic structures etc.
- English writing should be improved, particularly regarding syntax and style errors. Information Classification: General To address the above mentioned issues regarding literature review and future scope of the proposed study authors must add and cite some recent and well related manuscripts by Ashraf ul Quran, Tahir Mahmood and Muhammad Akram.

Is the work clearly and accurately presented and does it cite the current literature?

Partly

If applicable, is the statistical analysis and its interpretation appropriate?

Yes

Are all the source data underlying the results available to ensure full reproducibility?

No source data required

Is the study design appropriate and is the work technically sound?

Yes

Are the conclusions drawn adequately supported by the results?

Partly

Are sufficient details of methods and analysis provided to allow replication by others?

Yes

Reviewer Expertise:

NA

I confirm that I have read this submission and believe that I have an appropriate level of expertise to confirm that it is of an acceptable scientific standard, however I have significant reservations, as outlined above.

# Response

**Published:** 2025-01-08

**Authors:** Masresha Wassie Woldie

**Affiliations:** Bahir Dar University Department of Mathematics, Bahir Dar, Amhara, Ethiopia

1. we accept the title revision "New Approaches of Generalized Fuzzy Soft Sets on Fuzzy Codes and Its Applications on Decision-Makings” to New Approaches of Generalized Fuzzy Soft Sets on Fuzzy Codes and Its Applications in Decision-Makings”

 2. From definition 6 change by 12 from the reference Kong, Wang, and Wu 2011.

 3. The manuscript "Generalised" can't change by "Generalized". For example see the motivated papers "Generalised fuzzy soft set" and " Generalised interval-valued fuzzy soft set" etc
